# Positive Association between Blood 25-Hydroxyvitamin D Levels and Pterygium after Control for Sunlight Exposure

**DOI:** 10.1371/journal.pone.0157501

**Published:** 2016-06-10

**Authors:** Donghyun Jee, Eun Chul Kim, Eunyoung Cho, Jorge G. Arroyo

**Affiliations:** 1 Department of Ophthalmology and Visual Science, St. Vincent's Hospital, College of Medicine, Catholic University of Korea, Suwon, Korea; 2 Department of Ophthalmology and Visual Science, Bucheon St. Mary’s Hospital, College of Medicine, Catholic University of Korea, Bucheon, Korea; 3 Channing Division of Network Medicine, Brigham and Women's Hospital and Harvard Medical School, Boston, United States of America; 4 Department of Dermatology, The Warren Alpert Medical School of Brown University, Providence, United States of America; 5 Department of Ophthalmology, Beth Israel Deaconess Medical Center, Harvard Medical School, Boston, Massachusetts, United States of America; University of Alabama at Birmingham, UNITED STATES

## Abstract

**Purpose:**

To investigate the association between blood 25-hydroxyvitamin D levels and pterygium.

**Methods:**

Korean National Health and Nutrition Examination Survey 2008–2011 were used for the present epidemiologic study. A total of 19,178 participants aged ≥ 30 years were evaluated for blood 25-hydroxyvitamin D levels and performed ophthalmic slit lamp examinations. Pterygium was considered as a growth of fibrovascular tissue over the cornea.

**Results:**

The average blood 25-hydroxyvitamin D levels were 18.6 ng/mL, and prevalence of pterygium was 6.5%. The odds of pterygium significantly increased across blood 25-hydroxyvitamin D quintiles after controlling sun exposure time as well as other confounders such as sex, age, smoking, diabetes, hypertension (P < 0.001). The odds ratios (OR) for pterygium was 1.51 (95% Confidence Interval[95%CI]; 1.19–1.92) in the highest blood vitamin D quintile. Stratified analysis by sex showed a positive association between blood 25-hydroxyvitamin D levels and pterygium in both men (quintile 5 versus 1, OR; 1.68, 95%CI; 1.19–2.37) and women (quintile 5 versus 1, OR; 1.37, 95% CI; 1.00–1.88).

**Conclusions:**

Even after controlling sun light exposure time, we found a positive association between blood 25-hydroxyvitamin D levels and pterygium in a representative Korean population. The mechanism underlying this association is unknown.

## Introduction

Pterygium is a benign uncontrolled growth of conjunctiva. It can significantly disturb the visual function in advanced cases through irregular astigmatism, impaired tear film regularity, or visual occlusion by a large pterygium over the visual axis. In addition, associated inflammation can lead to conjunctival injection and ocular discomfort. Although the full pathophysiology of pterygium is unclear, ultraviolet-mediated limbal damage is a risk factor for initiation of pterygium [1, 2]. In addition, the development of pterygium involves epidermal proliferation [3], inflammatory infiltration [4], angiosis and fibrosis [5], and alteration in the epithelial-mesenchymal transition [6]. Recently, it has been reported that the S100 proteins, which are calcium-activated signaling proteins, may be associated with the formation of pterygium [7]. Pterygium tissue showed higher expression of S100 proteins than normal conjunctival tissue.

Vitamin D has not only function of calcium regulation but also other biologic functions such as anti-inflammation or anti-oxidation [8–10]. Vitamin D was inversely associated with chronic inflammation in many human studies [11]. Various ocular diseases including myopia [12], age-related macular degeneration [13], and diabetic retinopathy [14, 15] was found to be related with vitamin D. Our previous work demonstrated that vitamin D was inversely related with cataract [16], diabetic retinopathy [17], and age-related macular degeneration [18] in representative Korean population. In addition we reported no association between vitamin D and dry eye syndrome, which implicated the differential effect of vitamin D on ocular diseases [19].

However, epidemiologic studies on the association between vitamin D levels and pterygium are very limited. The results from our previous studies on the inverse association between blood vitamin D and cataract and age-related macular degeneration [17, 18] are interesting considering that 90% of vitamin D is generated in the skin through sunlight, which has been implicated as a risk factor for cataract and age-related macular degeneration [20–23]. Similarly, the mechanism underlying pathogenesis of pterygium includes sunlight exposure as a risk factor for pterygium [1, 2]. Thus, blood vitamin D levels have the possibility playing a role in pathogenesis of pterygium. In this study, the possible relationship between blood 25-hydroxyvitamin D levels and pterygium was evaluated in Korean adults. In addition, our result for pterygium was compared to the results of our previous reports about association between vitamin D and age-related macular degeneration, diabetic retinopathy, cataract, and dry eye syndrome.

## Methods

**Fig 1:**
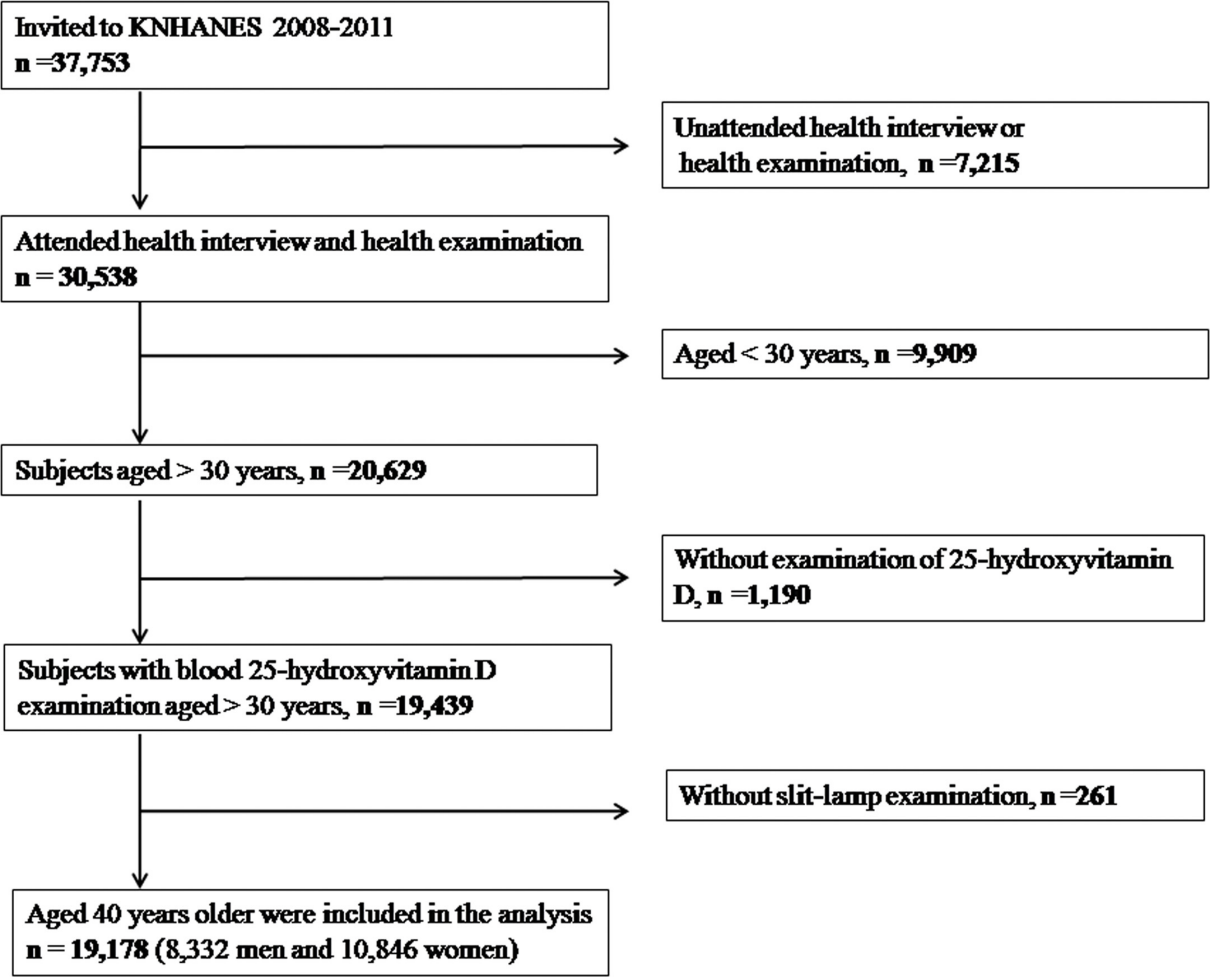
Flow diagram showing the selection of study participants.

The study design followed the tenets of the Declaration of Helsinki for biomedical research. Protocols for this study were approved by the institutional review board at the Catholic University of Korea in Seoul. All participants provided written informed consent. We used data from the Korean National Health and Nutrition Examination Survey (KNHANES). Details about the study design and the methods used have been reported elsewhere [24, 25]. KNHANES is a nationwide and population-based cross-sectional study. For the present study, we included data obtained from KNHANES 2008–2011. For the current study, 30,538 individuals who took part in KNHANES were enrolled. Of these, 9,909 participants aged <30 years, 1,190 participants without blood 25-hydroxyvitamin D levels, and 1,190 participants without information on the presence of pterygium were excluded from the study. Thus, 19,178 participants were used in the final analysis (Fig 1).

The analysis of blood 25-hydroxyvitamin D levels has been described elsewhere [26, 27]. A radioimmunoassay kit (DiaSorin Inc., Stillwater, MN, USA) was used for measurement of 25-hydroxyvitamin D levels using a gamma counter (1470 Wizard, Perkin-Elmer, Finland), followed by the standardization of vitamin D procedure [28]. Blood samples were collected after an 8-h fast, and they were transported to a laboratory of the Neodin Medical Institute after appropriate process. The measurement of 25-hydroxyvitamin D had the detection limit of 1.2 ng/ml. Interassay coefficients of variation were 2.8–6.2% for KNHANES 2008–2009, and 1.9–6.1% for for KNANES 2010–2011. A Hitachi 7600 clinical analyzer (Hitachi High-Technologies Corporation, Tokyo, Japan) was used for measurement of other clinical variables including total cholesterol, glucose, triglyceride and hemoglobin A1c levels.

The measurement of pterygium in KNHANES was documented in detail previously [29]. Briefly, slit-lamp eye examinations by a BQ 900^®^ (Haag-Streit AG; Koeniz, Switzerland) were used for participants. Pterygium was defined as a growth of fibrovascular tissue over the cornea. Other clinical and demographic characteristics were determined as follows. We calculated body mass indices by dividing weight (kg) by height (m)^2^. Diabetes mellitus was considered to be present when the subjects take anti-glycemic medication or a fasting blood-glucose level was more than126 mg/dL. Hypertension was considered to be present when subjects take antihypertensive medication or a systolic and diastolic blood pressure was more than 140 mmHg and 90 mmHg, respectively. Sunlight exposure time was evaluated by questionnaire whether subjects have sunlight exposure more than 5 hours per day or not. Smoking status was examined by questionnaire classifying participants into three categories: current, past, or non smokers.

The SPSS^®^ version 18.0 (SPSS, Chicago, IL, USA) were used for statistical analyses. Since KNHANES used stratified, multistage sampling method, we incorporated sampling weights as well as strata, sampling units in the statistical analysis. Continuous variables were presented with the mean and standard error (SE), and categorical variables were presented with the percentage and SE. To compare the patients’ demographic characteristics ANOVA or chi-square tests were used. We used logistic regression analyses after categorization of 25-hydroxyvitamin D levels into quintiles. To evaluate the confounding effect by confounders, we calculated three odds ratio (OR); the crude OR (Model 1), age and sex adjusted OR (Model 2), and sex, age, smoking, hypertension, diabetes, and sunlight exposure times adjusted OR (Model 3). We tested multicollinearity, and exclude variables which has a variance inflation factor more than 5 for the logistic regression analyses. *P* values less than 0.05 were regarded as statistical significance.

## Results

The average blood 25-hydroxyvitamin D levels were 18.6 ng/mL (SE, 0.1%). The prevalence of pterygium in both genders was 6.5% (SE, 0.3%). The prevalence of pterygium was 6.8% (SE, 0.3%) in men and 6.3% (SE, 0.3%) in women. Table 1 showed that participants with pterygium was significantly associated with old age, diabetes, hypertension, higher systolic blood pressure, higher fasting glucose levels, higher 25-hydroxyvitamin D levels, and higher sun-exposure times (*P* for all variables above < 0.001), compared with those without pterygium.

**Table 1:** Demographic and clinical characteristics, according to pterygium status in the Korean National Health and Nutrition Examination Survey 2008–2011.

Characteristics	No pterygium (n = 17630)	Pterygium (n = 1548)	*P*	Included (n = 19178)	no exam (n = 261)	*p*	Total (n = 19439)
**Male (%)**	49.7 (0.3)	51.9 (1.5)	.145	49.8 (0.3)	54.5 (3.4)	.170	49.9 (0.3)
**Age (yrs)**	48.9 (0.1)	61.7 (0.4)	< .001	49.7 (0.1)	49.2 (0.9)	.645	49.5 (0.5)
**Body mass index (kg/m2)**	23.8 (0.0)	23.9 (0.1)	.329	23.8 (0.0)	24.0 (0.2)	.549	23.9 (0.1)
**Systolic blood pressure (mmHg)**	119.3 (0.2)	126.5 (0.6)	< .001	119.8 (0.2)	121.0 (1.3)	.363	120.4 (0.7)
**Diastolic blood pressure (mmHg)**	77.7 (0.1)	78.4 (0.4)	.104	77.8 (0.1)	79.2 (0.9)	.124	78.5 (0.4)
**Fasting glucose (mg/dL)**	98.5 (0.2)	102.1 (0.9)	< .001	98.7 (0.2)	103.9 (2.2)	.023	101.3 (1.1)
**HbA1c (%)**	6.0 (0.0)	6.2 (0.1)	.012	6.1 (0.0)	6.1 (0.1)	.542	6.1 (0.1)
**Total cholesterol (mg/dL)**	190.9 (0.3)	192.3 (1.2)	.254	191.0 (0.3)	188.4 (2.6)	.342	189.7 (1.3)
**Triglyceride (mg/dL)**	142.3 (1.2)	143.7 (3.1)	.684	142.4 (1.1)	151.8 (8.7)	.282	147.1 (4.4)
**25-hydroxyvitamin D (ng/mL)**	18.5 (0.1)	20.4 (0.2)	< .001	18.6 (0.1)	18.0 (0.5)	.290	18.3 (0.3)
**Diabetes (%)**	9.8 (0.3)	14.6 (1.2)	< .001	10.1 (0.3)	12.2 (2.5)	.390	10.2 (0.3)
**Hypertension (%)**	31.0 (0.5)	48.4 (1.7)	< .001	32.1 (0.5)	33.9 (3.5)	.602	32.1 (0.5)
**Sun exposure (%)**			< .001			.715	
**> 5hrs/day**	19.6 (0.6)	35.1 (1.9)		20.6 (0.7)	19.3 (3.5)		20.6 (0.7)
**< 5hrs/day**	80.4 (0.6)	64.9 (1.9)		79.4 (0.7)	80.7 (3.5)		79.4 (0.7)
**Smoking status**			.059			.296	
**Never (%)**	52.2 (0.4)	52.6 (1.5)		52.2 (0.4)	47.3 (3.8)		52.2 (0.4)
**Former (%)**	12.0 (0.3)	14.6 (1.1)		12.2 (0.3)	11.1 (2.6)		12.2 (0.3)
**Current (%)**	35.8 (0.5)	32.8 (1.5)		35.6 (0.4)	41.6 (3.9)		35.7 (0.4)

Data are expressed as weighted means or weighted frequency (%) with standard errors.

As quintiles of blood 25-hydroxyvitamin D levels, subjects had a tendency to be male (*P* < 0.001), older (*P* < 0.001), hypertensive (*P* < 0.001), diabetic (*P* = 0.031), smoker (*P* < 0.001), and have higher fasting glucose (*P* = 0.010), higher total cholesterol (*P* = 0.006), and experienced longer sun exposures (*P* < 0.001, Table 2).

**Table 2:** Demographic and clinical characteristics by quintile blood 25-Hydroxyvitamin D categories among representative Korean adults aged 19 years or older.

Characteristics		Quartile blood 25-Hydroxyvitamin D level (ng/mL)				
	< 13.0	13.0–16.3	16.3–19.6	19.6–24.3	> 24.3	P for trend
**Number**	**3845**	**3833**	**3841**	**3841**	**3828**	
**Male (%)**	36.7 (0.9)	43.4 (1.0)	51.5 (0.9)	56.8 (1.0)	62.3 (0.9)	< .001
**Age (yrs)**	48.0 (0.3)	48.2 (0.2)	48.8 (0.3)	50.8 (0.3)	53.1 (0.4)	< .001
**Body mass index (kg/m**^**2**^**)**	23.5 (0.1)	24.0 (0.1)	23.9 (0.1)	24.1 (0.1)	23.7 (0.1)	< .001
**Systolic blood pressure (mmHg)**	118.6 (0.3)	118.7 (0.3)	119.3 (0.3)	120.6 (0.3)	121.8 (0.4)	< .001
**Diastolic blood pressure (mmHg)**	76.8 (0.2)	77.5 (0.2)	77.9 (0.2)	78.4 (0.2)	78.3 (0.2)	< .001
**Fasting glucose (mg/dL)**	98.1 (0.5)	97.7 (0.4)	99.7 (0.5)	98.7 (0.4)	99.5 (0.4)	.010
**HbA1c (%)**	6.1 (0.0)	5.9 (0.0)	6.1 (0.0)	6.0 (0.0)	6.2 (0.1)	.001
**Total cholesterol (mg/dL)**	189.0 (0.7)	190.7 (0.7)	191.9 (0.7)	193.2 (0.7)	190.0 (0.6)	< .001
**Triglyceride (mg/dL)**	143.3 (2.9)	146.4 (2.7)	140.5 (2.3)	143.1 (2.3)	138.4 (1.8)	.147
**Diabetes (%)**	9.8 (0.6)	8.8 (0.6)	10.4 (0.6)	10.1 (0.6)	11.1 (0.6)	.031
**Hypertension (%)**	29.3 (0.9)	29.9 (0.9)	30.4 (0.9)	34.3 (1.0)	37.2 (1.0)	< .001
**Sun exposure (>5hrs/day, %)**						< .001
**< 5hrs/day**	87.7 (0.7)	85.9 (0.8)	82.3 (0.9)	74.9 (1.2)	64.4 (1.6)	
**> 5hrs/day**	12.3 (0.7)	14.1 (0.8)	17.7 (0.9)	25.1 (1.2)	35.6 (1.6)	
**Smoking status**						< .001
**Never (%)**	60.3 (1.0)	55.8 (1.0)	52.3 (1.0)	48.0 (1.0)	43.7 (1.0)	
**Former (%)**	8.8 (0.6)	11.8 (0.7)	13.2 (0.6)	15.1 (0.8)	12.1 (0.8)	
**Current(%)**	30.9 (1.0)	32.3 (1.0)	34.6 (0.9)	36.9 (1.1)	44.1 (1.1)	

**Fig 2:**
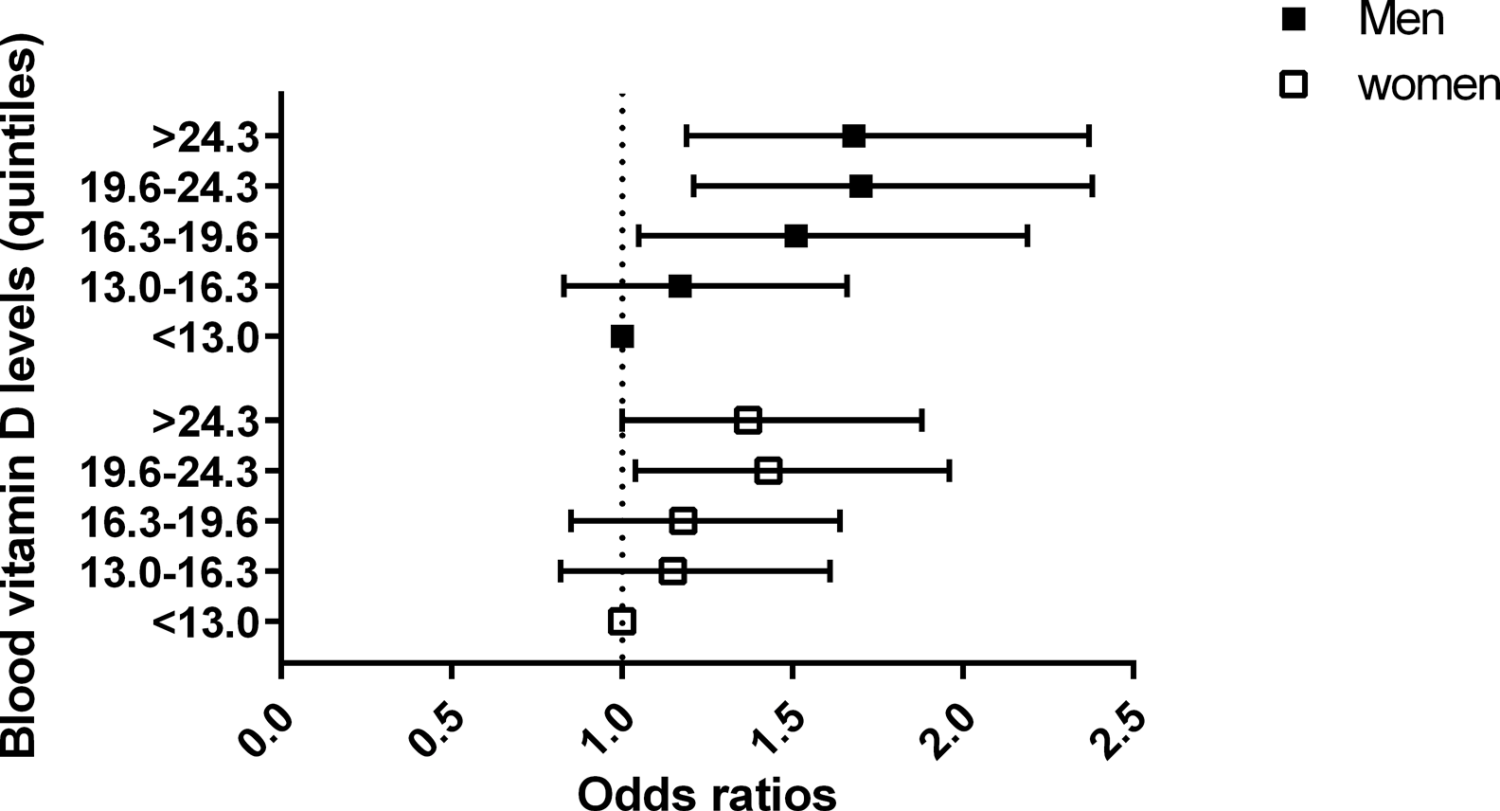
The odds ratios of pterygium according to quintiles of blood vitamin D levels (reference group = lowest vitamin D quintile group).

As the quintiles of the blood 25-hydroxyvitamin D levels increased, the odds of pterygium significantly increased (P < 0.001). Even after controlling potential confounders mentioned above, this positive association remained still strong and significant (P < 0.001, Table 3). OR for pterygium in highest quintile of blood 25-hydroxyvitamin D levels over lowest one is 2.20 (P < 0.001, 95%CI; 1.75–2.77). After controlling potential confounders, OR for pterygium in quintile 5 over quintile 1 is 1.51 (P < 0.001, 95%CI; 1.19–1.92, Fig 2). Stratified analysis by gender demonstrated that after adjusting for potential confounders, the association between higher blood 25-hydroxyvitamin D levels and the increasing odds of pterygium were significant both in men (quintile 5 versus 1, OR; 1.68, 95% CI; 1.19–2.37,) and women (OR; 1.37, 95% CI; 1.00–1.88,).

**Table 3:** Association between blood 25-hydroxyvitamin D and prevalence of pterygium among representative Korean adults.

Vitamin D quintiles (ng/mL)	Case/total number	Prevalence	Model 1	Model 2	Model 3
**Both gender**		**6.5 (0.3)**			
Quintile 1 (<13.0)	205/3845	4.4 (0.4)	1.00 (reference)	1.00 (reference)	1.00 (reference)
Quintile 2 (13.0–16.3)	243/3833	5.1 (0.4)	1.17 (0.94–1.47)	1.20 (0.96–1.52)	1.20 (0.95–1.22)
Quintile 3 (16.3–19.6)	291/3841	6.5 (0.5)	1.50 (1.20–1.88)*	1.49 (1.18–1.88)*	1.39 (1.10–1.75)*
Quintile 4 (19.6–24.3)	366/3841	7.8 (0.6)	1.84 (1.43–2.29)*	1.58 (1.26–1.98)*	1.48 (1.17–1.86)*
Quintile 5 (>24.3)	443/3828	9.2 (0.6)	2.20 (1.75–2.77)*	1.63 (1.29–2.06)*	1.51 (1.19–1.92)*
P for trend		< .001	< .001	< .001	< .001
**Men**		**6.8 (0.3)**			
Quintile 1 (<14.3)	91/1672	4.4 (0.5)	1.00 (reference)	1.00 (reference)	1.00 (reference)
Quintile 2 (14.3–17.7)	119/1666	5.3 (0.6)	1.21 (0.87–1.70)	1.23 (0.91–1.81)	1.17 (0.83–1.66)
Quintile 3 (17.7–21.1)	138/1665	6.8 (0.8)	1.61 (1.14–2.27)*	1.50 (1.05–2.14)*	1.51 (1.05–2.19)*
Quintile 4 (21.1–25.9)	186/1667	8.6 (0.8)	2.07 (1.51–2.83)*	1.78 (1.29–2.45)*	1.70 (1.21–2.38)*
Quintile 5 (>25.9)	211/1662	9.7 (0.9)	2.35 (1.70–3.25)*	1.78 (1.28–2.49)*	1.68 (1.19–2.37)*
P for trend		< .001	< .001	< .001	< .001
**Women**		**6.3 (0.3)**			
Quintile 1 (<12.2)	112/2169	4.5 (0.5)	1.00 (reference)	1.00 (reference)	1.00 (reference)
Quintile 2 (12.2–15.2)	130/2170	5.0 (0.5)	1.11 (0.82–1.51)	1.17 (0.84–1.62)	1.15 (0.82–1.61)
Quintile 3 (15.2–18.3)	144/2172	5.7 (0.6)	1.29 (0.94–1.75)	1.28 (0.93–1.76)	1.18 (0.85–1.64)
Quintile 4 (18.3–22.7)	189/2170	7.5 (0.7)	1.71 (1.26–2.31)*	1.57 (1.15–2.15)*	1.43 (1.04–1.96)*
Quintile 5 (>22.7)	228/2165	8.9 (0.8)	2.07 (1.54–2.79)*	1.53 (1.13–2.08)*	1.37 (1.00–1.88)*
P for trend		< .001	< .001	.001	.016

Prevalence was expressed as weighted estimates [%] (standard errors [%], 95% confidence intervals).

Model 1: Crude odds ratios. Model 2: adjusted for sex and age. Model 3: adjusted for sex, age, diabetes, hypertension, sunlight exposure time, smoking, and body mass index.

* p < 0.05

## Discussion

Our study is the first to evaluate the association between blood 25-hydroxyvitamin D levels and pterygium. We found that even after adjusting for the sun light exposure time, the adjusted odds of pterygium was associated with the increasing quintiles of the blood 25-hydroxyvitamin D levels, and blood 25-hydroxyvitamin D levels were positively associated with of the prevalence of pterygium. Based on this unexpected result we hypothesized that blood vitamin D levels have an inverse association with the prevalence of pterygium.

The exact mechanism underlying this relationship is unknown. One possible explanation is that high vitamin D levels may elevate blood calcium levels and activate a calcium-activated signaling protein, S100 protein, which has been implicated as a cause of pterygium development [7]. Many pathophysiology in pterygium development including angiogenesis, transdifferentiation, and cellular proliferation may be contributed to calcium signaling activities [2]. A recent in vitro study demonstrated that calcium-free bathing medium made from blood reduced the number of pterygium-derived fibroblasts, which is the main causal cell in pterygium development [30]. In addition, suppressed calcium signaling activity reduced the growth rates of pterygian-derived fibroblasts [30]. It suggests that the calcium store plays important role in pathophysiology of pterygian-derived fibroblasts. Thus, a higher level of vitamin D may elevate the cellular calcium levels, which would enhance the calcium signaling development of pterygium through S100 protein. However, we could not assess the blood calcium levels or S100 protein levels. Further studies are required to identify the relationship between blood calcium levels and pterygium.

Another possible explanation is the residual confounding factor of sun exposure on the association between vitamin D and pterygium. Because the majority of vitamin D is synthesized in the skin from sunlight, the subjects with high blood vitamin D levels could have experienced longer sun exposure times. Although we adjusted for sun exposure time in model 3, it is a dichotomous variable (≥ 5 h or < 5 h/day). There is a possibility that sun exposure is a residual confounding factor. However, the association between vitamin D and pterygium was consistently strong both before and after adjusting for sun exposure time. Thus, it is unlikely that the residual confounding factor of sun exposure time would cause the strong positive association between blood vitamin D and pterygium. The contribution of sun exposure time is further supported by a comparison of our results with the results of previous studies involving age-related macular degeneration and cataract, in which sunlight exposure was an established risk factor [16–19].

**Fig 3:**
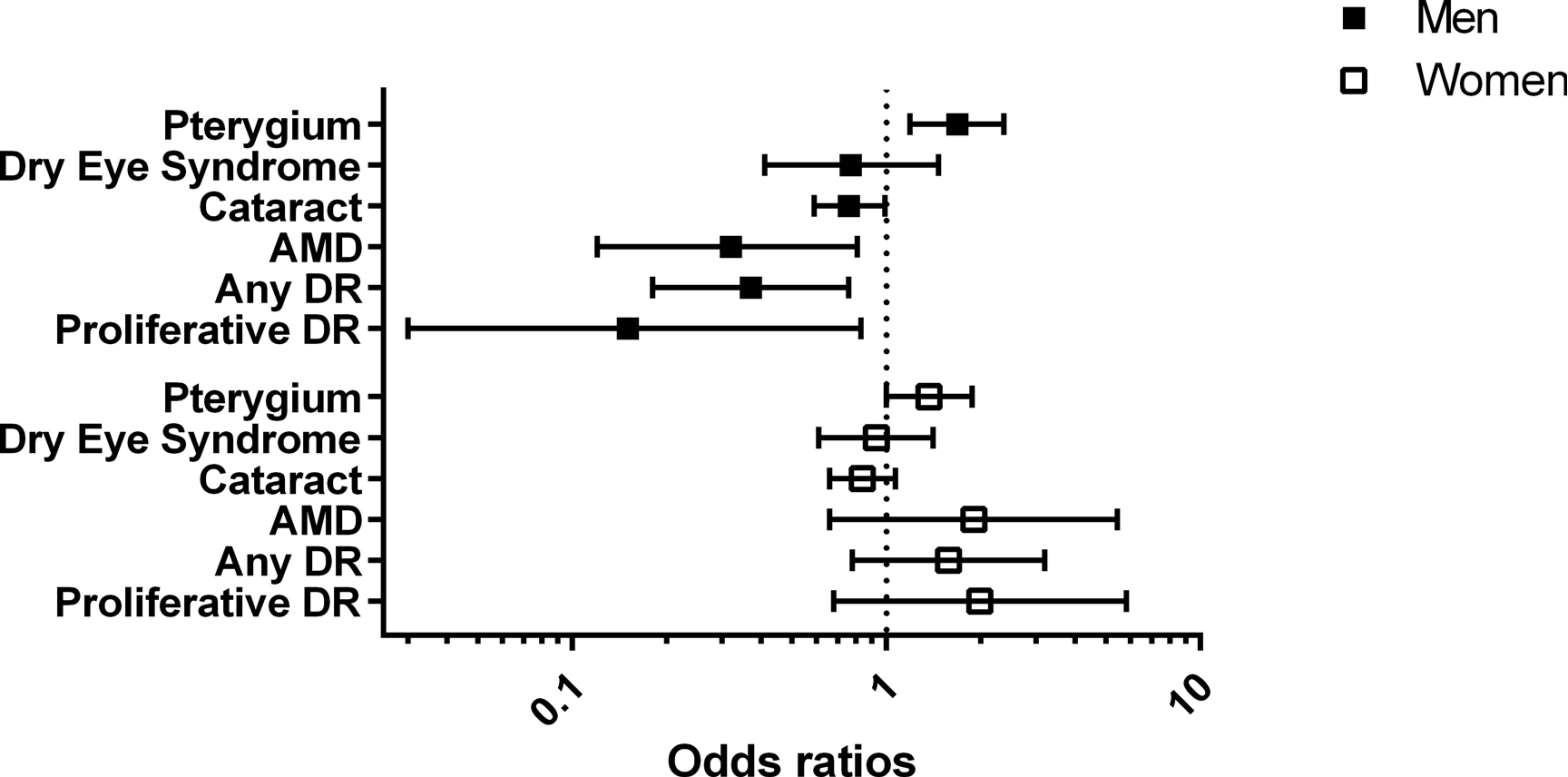
The comparison of odds ratios of ocular diseases including dry eye syndrome (DES), cataract, age-related macular degeneration (AMD), any diabetic retinopathy (DR), and vision-threatening DR (VTDR) according to the blood vitamin D levels (reference group = lowest vitamin D quintile group).

We compared the association of pterygium with those for four other ocular diseases (diabetic retinopathy, age-related macular degeneration, cataract, and dry eye syndrome) from our previous reports which have used the same KNHANES population (Fig 3) [16–19]. The blood vitamin D levels were inversely associated with the ocular diseases, although the strength of association was different among the ocular diseases. In men, the ORs of late age-related macular degeneration, diabetic retinopathy, cataract, and dry eye syndrome were 0.32 (95% CI, 0.12–0.81), 0.37 (95% CI, 0.18–0.76), 0.76 (95% CI, 0.59–0.99), and 0.85 (95% CI, 0.55–1.30), respectively. However, in the present study, the blood vitamin D levels were positively associated with pterygium (OR = 1.68, 95% CI = 1.19–2.37). Moreover, the association between vitamin D and pterygium was stronger than those of other diseases, given that the relative odds of pterygium in those with 3rd, 4th,and 5th vitamin D quintiles versus the lowest one were significantly increased, whereas relative odds of other ocular diseases in those with only 5th vitamin D quintile versus lowest one was significantly decreased. In addition, the association between vitamin D and pterygium was shown in both men and women, whereas the association between vitamin D and other ocular diseases has been shown only in men, not women. These comparisons imply that the underlying mechanism of association between blood vitamin D and pterygium may be different from those of the association between blood vitamin D and other diseases (diabetic retinopathy, age-related macular degeneration, cataract, and dry eye syndrome) in our previous reports [16–19].

The average vitamin D concentration (18.6 ng/mL) was low and in the range indicating mild to moderate vitamin D insufficiency in clinical guidelines. These findings are supported by a previous study of Korea, in which prevalence of vitamin D insufficiency was 47.5% in men, and 64.5% in women [31]. In addition, young adults aged 20–29 years showed the prevalence of vitamin D insufficiency 65.0% in men and 79.9% in women. It implicates the vitamin D insufficiency could be a greater threat to younger generation in Korea.

The present study has both strength and limitations. Strength is the large number of participants in the present study. Another strength is the study’s design of nation-wide survey with stratified, multi-clustered sampling. Limitation of this study is that seasonal variations of vitamin D levels were not considered. Unfortunately, KNHANES does not have information on sampling season. A recent study showed that an Asian population did not display any significant seasonal variation in vitamin D status [32]. However, another study reported significant seasonal variation with lower vitamin D levels in winter [33]. Another limitation is that our study measured only 25-hydroxyvitamin D levels, which may not sufficient to reflect the body vitamin D levels. The current dogma is that vitamin D is activated by 25-hydroxylation and then 1,25-hydroxylation. Recently, novel pathways of vitamin D3 were found [34]. Slominski et al discovered the novel sequential hydroxylation that starts at carbon-20, which is initiated by CYP11A1.[35–38] Predominant pathway is from vitamin D3 through 20-hydroxyvitamin D to 20,23-hydroxyvitamin D.[37] Finally, our study design is a cross-sectional study, which introduced difficulties in reasoning causality.

In conclusion, our study is the first analysis of population-based epidemiologic data on the association between blood 25-hydroxyvitamin D levels and pterygium. We found a positive association between blood 25-hydroxyvitamin D levels and pterygium even after adjusting for the confounder of sun light exposure time, which is contrary to the results of our previous studies. The mechanism underlying this association is unknown and warrants further study.
